# Fatty acids and selected endocannabinoids content in cerebrospinal fluids from patients with neuroinfections

**DOI:** 10.1007/s11011-018-0347-7

**Published:** 2018-12-05

**Authors:** Jacek Czepiel, Joanna Gdula-Argasińska, Grażyna Biesiada, Beata Bystrowska, Artur Jurczyszyn, William Perucki, Katarzyna Sroczyńska, Anna Zając, Tadeusz Librowski, Aleksander Garlicki

**Affiliations:** 1 Department of Infectious and Tropical Diseases, Jagiellonian University Medical College, Krakow, Poland; 2 Department of Radioligands, Faculty of Pharmacy, Jagiellonian University Medical College, Medyczna 9, 30-688 Krakow, Poland; 3 Chair of Toxicology, Faculty of Pharmacy, Jagiellonian University Medical College, Krakow, Poland; 4 Department of Hematology, Jagiellonian University Medical College, Krakow, Poland; 5 Department of Medicine, John Dempsey Hospital, University of Connecticut, Farmington, CT USA

**Keywords:** Cerebrospinal fluid, Inflammation, Pathogenesis, Fatty acids, Endocannabinoids, Neuroinfections

## Abstract

Neuroinfections are a significant medical problem and can have serious health consequences for patients. Their outcome, if not fatal, can be associated with permanent residual deficits. Cerebrospinal fluid (CSF) examination is commonly used for meningitis confirmation. Fatty acids (FA) are precursors of lipid mediators with pharmacological activity. They actively modulate inflammation as well as contribute to its resolution. Therefore the aim of this study was to determine the FA and selected endocannabinoids (ECB) content in the CSF obtained from patients with bacterial (BM) and viral meningitis (VM) using chromatographic techniques. A significantly lower level of saturated FA was found in patients with BM and VM as compared to controls. There was a significantly higher concentration of long-chain monounsaturated FA and polyunsaturated n-6 FA in the CSF obtained from patients with neuroinfection. Moreover, a significant reduction of n-3 FA in CSF obtained from patients with BM and VM was demonstrated. The highest amount of ECB was detected in the CSF of patients with VM: eicosapentaenoyl ethanolamide (1.65 pg/mL), docosahexaenoyl ethanolamide (655.5 pg/mL) and nervonoyl ethanolamide (3.09 ng/mL). Results indicate the participation of long-chain monounsaturated and polyunsaturated FA and their derivatives in the inflammatory process and likely in the process of resolution of inflammation during neuroinfection. It seems that the determination of the FA and ECB profile in CSF may be a valuable biomarker of health and may allow the development of new pharmacological strategies, therapeutic goals and fatty acids supplementation necessary in the fight against inflammation of the central nervous system.

## Introduction

Despite progress in the prevention and treatment of infectious diseases, central nervous system (CNS) infections are a persistent problem in modern medicine. Neuroinfections account for significant worldwide morbidity and mortality, and are observed throughout the globe in every age group from neonates to older adults. They are characterized by a variety of symptoms ranging from fever and headaches to the most serious consequences including increased intracranial pressure and irreversible damage to the nervous system, which, if not fatal, can be associated with permanent residual deficits (Leibovitch and Jacobson 2016).

Fatty acids (FA) and their derivatives are important mediators in the human immune system. The intensity of the inflammatory process is influenced by changes in the metabolism of eicosanoids, endocannabinoids (ECB) and other lipid mediators (Stillwell and Wassall 2003; Shaikh and Edidin 2008; Serhan and Petasis 2011; Calder 2016).

CNS is an isolated place for infection, protected by the blood-brain barrier (BBB). The BBB provides structural as well as functional protection by maintaining homeostasis of the CNS microenvironment, protecting the nervous system against harmful factors, and also allowing selective transport of substances between blood and cerebro-spinal fluid (CSF). The BBB is therefore a barrier to pathogens, but also creates unique conditions for the operation of the host immune system. Selective BBB permeability is associated with a different structure of the capillary endothelium, forming a physical barrier. Endothelial cells are connected with each other through adherens junctions (AJs) and tight junctions (TJs), which have no gaps or pores, therefore substances present in the plasma can overcome the endothelium barrier only by means of transcellular transport (Ballabh et al. 2004; Nakagawa et al. 2009; van Sorge and Doran 2012).

The few studies of FA concentration in human CSF concerned only cases of strokes and brain injuries (Pilitsis et al. 2001, 2003a, b). The concept of these studies resulted from the previously shown significant increases of FA during pathological processes in nervous system, mainly ischemia in animal studies (Yasuda et al. 1985; Abe et al. 1987; Yoshida et al. 1980). Pilitsis et al. (2003a) assessed concentrations of arachidonic, docosahexaenoic, linoleic, myristic, oleic and palmitic acids in CSF from patients with ischemic and primary intracerebral hemorrhagic stroke (Pilitsis et al. 2003a). The concentrations of all studied FA were significantly greater than in control patients. Moreover, the higher concentration of polyunsaturated fatty acids (PUFA) significantly correlated with the worse Glasgow Coma Scale (GSC) score on admission and with the final outcome of stroke (Pilitsis et al. 2003a). Similar results were obtained by the authors who compared FA in people with traumatic brain injury compared to healthy people, the concentrations of arachidonic, DHA, myristic, oleic, palmitic, linoleic were significantly greater among patients with brain injury. There were no significant differences in FA concentration depending on Glasgow Outcome Scores (GOS), however, in patients who had early clinical improvement, AA, DHA and linoleic acids were significantly lower than in patients with worse outcome ratings. Pilitsis et al. (2003b) suggested that concentrations of FA may be useful as predictive markers of outcome in traumatic brain injury patients (Pilitsis et al. 2003b). Similar results for FA after brain injuries were observed in rat studies (Homayoun et al. 2000).

So far, the role of FA and their derivatives in the course of infection has been demonstrated (Shaikh and Edidin 2008; Tiesset et al. 2009; Czepiel et al. 2016), but there are no studies assessing this effect during neuroinfection. Moreover, the concentration of FA and ECB in neuroinfections was never directly assessed in the material taken from the CNS.

Taking this into account, the aim of our work was to assess the FA and ECB content in CSF during various etiologies of infection of the nervous system.

## Materials and methods

### Patients

The study group included 57 patients: 19 patients with bacterial meningitis (BM) (6 women and 13 men), 19 patients with viral meningitis (VM) (9 women and 10 men), and 19 patients (8 women and 11 men) in whom meningitis was excluded, who were hospitalized at the Department of Infectious and Tropical Diseases, Jagiellonian University Hospital in Krakow, Poland. All patients had signed a filled informed consent to participate in the examination.

The diagnosis of meningitis was based on history, physical examination and laboratory testing. Meningitis was confirmed or excluded after CSF examination, in which cytosis and the level of protein, glucose and white blood cells (WBC) were assed. Blood tests included WBC with differential leukocyte count and C-reactive protein (CRP), the tests were performed according to generally accepted standard methods. Moreover, each sample of CSF underwent microbiological assessment. Exclusion criteria included any other acute or chronic inflammatory diseases. Samples from each group were obtained from lumbar punctures for medically indicated reasons. All samples came from CSF left over in the main laboratory. Blood tests, demographic and clinical information were recorded using standardized data collection forms. The study was conducted in accordance with the Declaration of Helsinki 1975, and approved by the Jagiellonian University Ethics Committee (approval no 1072.6120.52.2018).

### Lipid extraction

One mL of CSF were acidified with 50 μL of 0.01% formic acid (Sigma-Aldrich, St. Louis, MO, USA). Ten microliter of 0.001% butylated dihydroxytoluene (BHT, Sigma-Aldrich) was added to prevent lipid oxidation. Lipids were extracted with chloroform–methanol solution (2:1 *v*/v) (Merck, Darmstadt, Germany). Two microliters of internal standards were used throughout: eicosapentaenoyl ethanolamide (EPEA-d4) and docosahexaenoyl ethanolamide (DHEA-d4), at concentration 10 μg/mL (Cayman Chemical, Ann Arbor, MI, USA). Samples were vortexed for 30 s and centrifuged for 10 min at 1000 x g. Organic phases were collected and dried under a stream of nitrogen. The residue was dissolved in 40 μL of acetonitrile (Merck).

### Fatty acid analysis

The synthesis of fatty acid methyl esters (FAME) was carried out with 14% BF_3_ in methanol. FAME were analyzed using gas chromatography (Agilent 6890 N, Agilent Inc., Santa Clara, CA, USA) with a J&W DB-23 (60 m, ID 0.25 mm, 0.25 μm) column, as described earlier (Gdula-Argasińska et al. 2016; Gdula-Argasińska and Bystrowska 2016). FAME were identified according to standards (Sigma–Aldrich). The data were analyzed using ChemStation. Results were expressed as relative percentages of the sum of saturated (SFA), monounsaturated (MUFA), n-3 and n-6 FA.

### LC-MS/MS conditions

Liquid chromatography was performed using an Agilent 1100 LC system (Agilent Technologies, Waldbronn, Germany). Chromatographic separation was carried out with a Thermo Scientific BDS HYPERSIL C18 column (100 × 3 mm I.D., 3 μm particle size). The advance column, with its pre-column (100 × 3 mm I.D., 3 μm particle size), was set at 40 °C with a mobile phase flow rate of 0.3 mL/min. The gradient elution mobile phases consisted of formic acid (0.02 mol/L) in acetonitrile and formic acid (0.02 mol/L) in water. MS/MS analyzes were performed on an Applied Biosystems MDS Sciex (Concord, Ontario, Canada) API 2000 triple quadruple mass spectrometer equipped with an electrospray ionization (ESI) interface. ESI ionization was performed in positive ionization mode. The multiple reaction monitoring (MRM) mode of the dominant product ion for each ECBs was used. A comparison of the paired ion (precursor and product ion m/z values) and LC retention times with standards served to confirm the identification of ECBs in the samples. Arachidonoyl ethanolamine (AEA), AEA-d4, 2-ariachonoylglycerol (2-AG), eicosapentaenoyl ethanolamide (EPEA), EPEA-d4, docosahexaenoyl ethanolamide (DHEA), DHEA-d4 and nervonoyl ethanolamide (NEA) standards were purchased from Cayman Chemical. An ion pair was 348 > 62 for AEA, 379 > 269 for 2-AG, 346 > 62 for EPEA, 372 > 62 for DHEA, 409 > 62 for NEA, 350 > 62 for EPEA-d4 and 376 > 66 for DHEA-d4. Data acquisition and processing were performed using Applied Biosystems Analyst version 1.4.2 software as described previously (Gdula-Argasińska and Bystrowska 2016).

### Statistics

All data are presented as means ± standard deviation (SD) or medians and lower (Q_25_) and upper (Q_75_) quartiles. Normal distribution of variables was checked using the Levene test. Differences between study groups were determined using the one-way ANOVA and Scheffe post hoc test, or Kruskal-Wallis test if normality was not observed. Analysis of similarities between content of the FA in CSF of studied groups was performed using clustering methods to identify homogenous groups of cases. Ward’s method with Euclidean distance matrix was used to join objects into clusters such that the variance within a cluster is minimized and to maximize the significance of differences between clusters. Calculations were performed using STATISTICA 13 (StatSoft Inc., Tulsa, OK, USA), and statistical significance was defined as *p* ≤ 0.05.

## Results

### Markers of inflammation

Biochemical tests show significant differences between the analyzed groups in the areas of CRP, WBC and CSF parameters. Patients with BM were characterized by the highest values ​​of markers of inflammation and lowering of glucose in CSF. In the group of people with VM the markers of inflammation were slightly raised, and the glucose concentration in CSF was normal. Patients in the control group, as predicted, were characterized by correct values ​​of the parameters analyzed (Table 1).

**Table 1:** Comparison of WBC, CRP and biochemical CSF parameters in the analyzed groups. Medians, lower and upper quartiles, *n* = 19

Parameter	BM (group 1)		VM (group 2)		Control group (group 3)		*p*
	Median	Q_25_-Q_75_	Median	Q_25_-Q_75_	Median	Q_25_-Q_75_	
CRP [mg/L]	78	34–160	3.2	2–26	1.2	1–5	1*vs*2 = 0.000 1*vs*3 = 0.04 2*vs*3 = 0.000
WBC [×10^3^/μL]	11.4	7.7–22.1	7.8	5.4–9	5.2	4.7–6.3	1*vs*2 = 0.005 1*vs*3 = 0.01 2*vs*3 = 0.000
CSF pleocytosis [count /μL]	1150	237–1600	62	18–150	2	1–4	1*vs*2 = 0.000 1*vs*3 = 0.000 2*vs*3 = 0.000
CSF protein [g/L]	3.2	1.2–5.2	0.6	0.5–0.8	0.4	0.3–0.6	1*vs*2 = 0.000 1*vs*3 = 0.005 2*vs*3 = 0.000
CSF glucose [mmol/L]	1.6	0–2.1	2.7	2.5–3.3	3.1	2.9–3.4	1*vs*2 = 0.000 1*vs*3 = 0.02 2*vs*3 = 0.000

*BM* bacterial meningitis, *CRP* C-reactive protein, *CSF* cerebro-spinal fluid, *Q*_*25*_ lower quartile, *Q*_*75*_ upper quartile, *VM* viral meningitis, *WBC* white blood cells

### Fatty acids content in CSF

FA composition in patients with neuroinfections and controls are shown in Table 2. The saturated fatty acids (SFA) index in CSF from VM patients (30.7%) was statistically lower than in BM patients (35.01%), and also when compared to control (39.14%). The monounsaturated fatty acid (MUFA) index in CSF from controls was significantly higher (35.51%) than both BM (24.52%) and VM (26.46%). We noticed changes among n-3 and n-6 ​​FA. Total n-3 FA were significantly decreased, and total n-6 FA were significantly increased in the CSF of patients with meningitis compared to healthy persons. The CSF n-3/n-6 ratio was significantly decreased (more than three-fold) in meningitis patients compared to the control group (Table 2). There was no significant difference in FA between BM and VM groups. The general trend of changes in both groups with neuroinfection was both the same and with similar intensity. We did not show statistically significant differences between the BM and VM groups in the n-3 FA, n-6 FA and n-3/n-6 ratio. An increase in monounsaturated and polyunsaturated n-3 long-chain FA in the CSF obtained from patients with neuroinfection was observed. In patients with VM, the amount of linoleic acid increased more than 2.5 times, while in patients with BM there was statistically significant increase (three times) in the content of docosahexaenoic acid (DHA, C22: 6 n-3) in CSF.

**Table 2:** Fatty acid content [%] in cerebrospinal fluid from healthy, bacterial and viral meningitis. Means ± SD; n = 19

FA	BM	VM	Control
C8:0	2.28^#^ ± 0.36	1.15* ±0.26	1.64 ± 0.26
C10:0	5.43^#^ ± 1.14	3.51^#,^* ± 0.83	1.70 ± 0.83
C12:0	2.72^#^ ± 0.71	1.26^#,^* ± 0.51	5.67 ± 0.51
C14:0	2.10 ± 0.46	1.94 ± 0.34	2.74 ± 0.34
C16:0	8.27^#^ ± 1.27	9.72 ± 0.92	12.28 ± 0.92
C18:0	13.31 ± 1.94	12.66^#^ ± 1.41	17.96 ± 1.41
C24:0	0.91^#^ ± 0.38	0.52* ±0.27	0.30 ± 0.27
SFA	35.01 ± 6.27	30.70^#,^* ± 4.55	39.14 ± 4.55
C14:1n-5	1.95^#^ ± 0.40	2.20^#^ ± 0.29	0.16 ± 0.29
C16:1n-7	1.43^#^ ± 0.39	1.99* ±0.29	2.18 ± 0.29
C18:1 n-9	19.07^#^ ± 2.80	21.35^#^ ± 2.03	32.75 ± 2.03
C20:1n-9	1.07^#^ ± 0.36	0.52^#,^* ± 0.26	0.26 ± 0.00
C22:1n-9	0.34^#^ ± 0.11	0.19^#,^* ± 0.08	0.08 ± 0.00
C24:1n-9	0.66^#^ ± 0.12	0.21^#,^* ± 0.09	0.09 ± 0.00
MUFA	24.52^#^ ± 4.17	26.46^#^ ± 3.03	35.51 ± 2.61
C18:1n-9 t	3.33^#^ ± 1.15	0.72* ±0.84	0.72 ± 0.84
C18:2n-6 t	0.51 ± 0.25	0.47^#^ ± 0.18	0.78 ± 0.18
*trans*	3.84^#^ ± 1.41	1.19 ± 1.02	1.50 ± 1.02
C18:2n-6	2.63^#^ ± 0.71	4.34* ±0.52	5.03 ± 0.52
C18:3n-6	5.00^#^ ± 2.89	12.40^#,^* ± 2.09	2.09 ± 0.26
C20:2n-6	3.22^#^ ± 1.32	2.85^#^ ± 0.96	0.96 ± 0.05
C20:3n-6	4.58^#^ ± 1.21	2.96^#,^* ± 0.88	0.60 ± 0.88
C20:4n-6	5.51 ± 1.26	5.38 ± 0.92	4.99 ± 0.92
C22:2n-6	9.51^#^ ± 2.60	8.07^#^ ± 1.89	0.11 ± 1.89
n-6	30.46^#^ ± 10.00	35.99^#^ ± 7.25	13.78 ± 4.52
C18:3n-3	2.02^#^ ± 0.72	2.00^#^ ± 0.52	3.32 ± 0.52
C20:3n-3	0.80 ± 0.44	0.79 ± 0.32	0.99 ± 0.32
C20:5n-3	2.30^#^ ± 0.66	2.52^#^ ± 0.48	5.42 ± 0.48
C22:6n-3	1.04^#^ ± 0.37	0.36* ±0.27	0.34 ± 0.27
n-3	6.16^#^ ± 2.20	5.67^#^ ± 1.60	10.07 ± 1.60
n-3/n-6	0.20^#^ ± 0.02	0.22^#^ ± 0.06	0.73 ± 0.15

*BM* bacterial meningitis, *FA* fatty acids, *MUFA* monounsaturated fatty acids, *SFA* saturated fatty acids, *VM* viral meningitis; #*p* < 0.01 vs. control, *p < 0.01 vs. BM

**Fig. 1:**
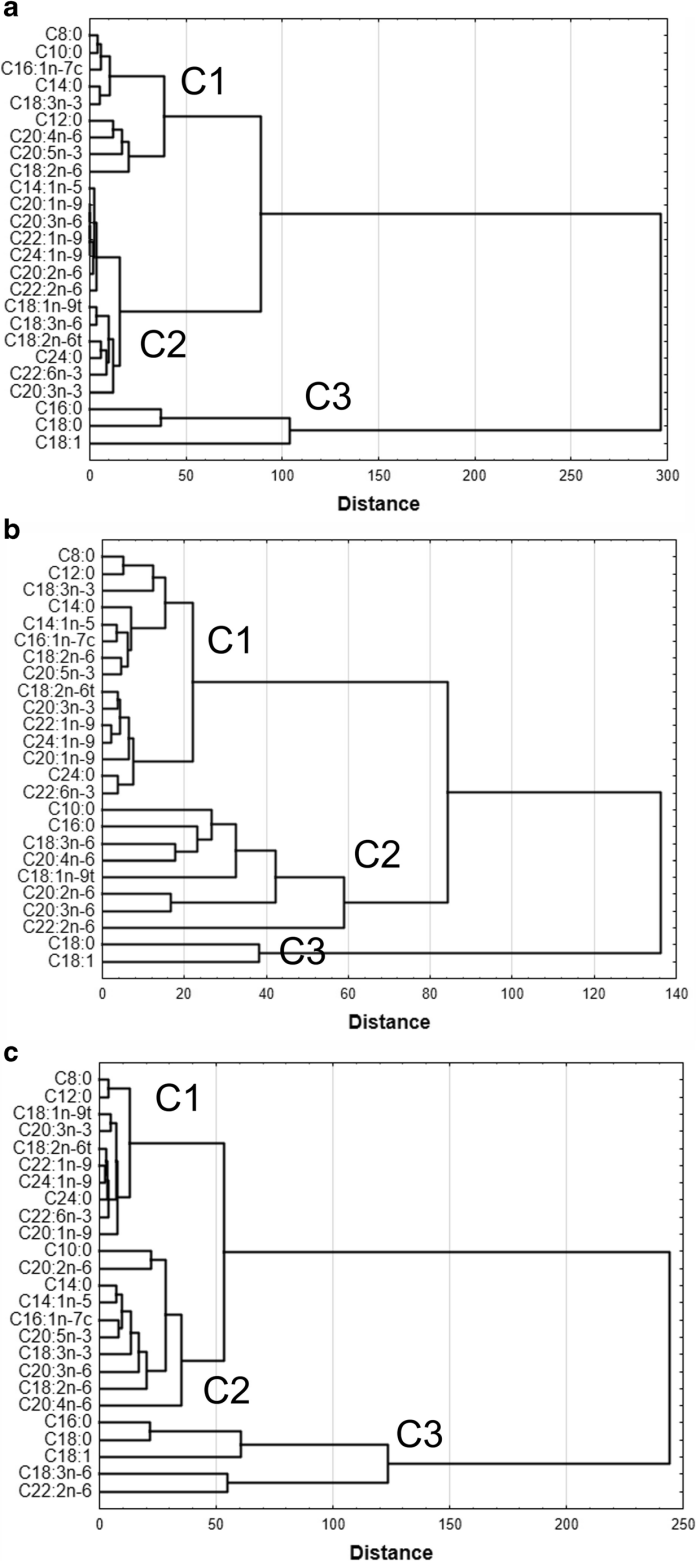
Cluster analysis of the FA profile in CSF from healthy people (**a**) and from patients with bacterial (**b**) and viral malignance (**c**). Data were segregated into three unique clusters of variables by hierarchical cluster analysis

To determine the covariant behavior of the measured variables, we analyzed such variables to identify which one best discriminates the FA profile in CSF from healthy people and patients with BM and VM (Fig. 1a–c). Variables were separated into three unique clusters. In healthy patients, CSF cluster 1 consisted of SFA (C8:0, C10:0, C12:0, C14:0) and C16:1 as well as C18:3 n-3, EPA and C18:2 n-6 and arachidonic acid (AA, C20:4 n-6). Cluster 2 consisted of a long chain of MUFA (C22:1 and C24:1), trans FA (C18:1 t, C18:2 t), C24:0, DHA and n-6 ​​FA (18:3 n-6, 20:3 n-6). In contrast, variants 16:0, 18:0, and 18:1 grouped further away (Fig. 1a).

In patients with BM, in contrast to the control, grouping of n-3 FA in the first cluster consisted of the following variants: 18:2 n-6, 18:3 n-3, EPA, DHA and saturated fatty acids C8:0, C12:0, C14:0 as well as long chain monounsaturated FA. n-6 and trans FA variants dominated in the second cluster (C18:3 n-6, C20:2 n-6, C20:3 n-6, AA and C22:6 n-6. In cluster 3, we noted grouping of C18:0 and C18:1 FA (Fig. 1b). In CSF from patients with VM in the cluster 1 long chain FA were present (C20:3 n-3, C22:1, C24:1, C24:0, C22:6 n-6, C20:1) as well as saturated FA (C8:0 and C12:0). Cluster 2 consists of SFA (C10:0, C14:0), MUFA (C14:1, C16:1), C18:3 n-3 as well as n-6 FA (C18:2 n-6, C20:3 n-6 and AA). In the cluster 3 the following FA were present: C16:0, C18:0, C18:1 and n-6 FA (C18:3 n-6, C22:6 n-6) (Fig. 1 c).

### Endocannabinoids content

Arachidonoyl ethanloamide and 2-ariachonoylglycerol was not detected in CSF of all groups. Statistically the highest concentration of EPEA (1.65 pg/mL), DHEA (655 pg/mL) and NEA (3.09 ng/mL) were detected in CSF from VM patients when compared to control (Table 3). In the samples from BM patients the ECB concentrations were lower than in VM, but significant differences were observed only for DHEA content (Table 3).

**Table 3:** Endocannabinoids content in CSF of BM, VM patients and healthy control. Medians, lower and upper quartiles, n = 19

ECB	BM (group 1)		VM (group 2)		Control group (group 3)		*p*
	Median	Q_25_-Q_75_	Median	Q_25_-Q_75_	Median	Q_25_-Q_75_	
EPEA [pg/mL]	0.5	0.3–1.9	1.6	0.5–4.3	0.4	0.3–0.5	2*vs*3 = 0.01
DHEA [pg/mL]	139	109–168	655	169–840	130	100–144	1*vs*2 = 0.002 2*vs*3 = 0.000
NEA [ng/mL]	1.5	0.8–3.5	3.1	1.5–4.1	1.6	1.0–2.2	2*vs*3 = 0.022

## Discussion

In our study for the first time we showed significant changes in the composition of fatty acids and endocannabinoids in the course of neuroinfections of various etiologies. Until now, the assessment of FA composition in CSF has been evaluated in few studies, but the evaluation has never been carried out in the course of neuroinfection. It was not known whether FA as well as ECB in the CNS during infection play a similar role as has been shown during infection in other parts of the body. Our study again demonstrates the important role of FA and their derivatives in the course of central nervous system pathology. There are hypothetically two mechanisms which could explain the changes in CSF composition observed in our study.

### Fatty acids contribute to the systemic and local immune response

Polyunsaturated fatty acids are essential for brain homeostasis and functions and are the main components of neuronal membranes (Kong et al. 2011; Calder 2016). FA metabolism may be an important mechanism underlying rapid neuroinflammation in pathologic processes such as neuroinfections.

The first mechanism would result from the fact that FA contribute to the systemic and local immune response triggered by pathogens that reached the CNS, as such changes in FA as well as in ECB in CSF would be a reflection of the ongoing inflammatory process in the CNS. In our study, we showed a significant increase in n-6 FA in the course of neuroinfection compared to the control group, this effect occurred regardless of etiology. The strongest increase of n-6 FA, mainly C18:3 (gamma-linolenic acid, GLA), C20:2 (dihomo-gamma linolenic acid, DGLA), C20:3, C20:4 (arachidonic acid, AA) and C22:2 (adrenic acid) was observed in CSF from VM patients. DGLA, which is product of GLA conversion by elongase 5 (ELOVL5), can be enzymatically transformed to several metabolites with anti-inflammatory properties (Sergeant et al. 2016). DGLA may be also a precursor of arachidonic acid (AA). As a result of AA enzymatic oxidation, prostaglandins, leukotrienes, and lipoxins are formed and may generally promote inflammation (Serhan and Petasis 2011; Norris and Dennis 2014). Results obtained in our study suggest that n-6 FA significantly attenuate in an neuro-inflammatory responses. We also observed cluster characteristic grouping of n-6 fatty acids in patients with BM and VM. Similar to the changes in the n-6 FA in both neuroinfection groups, there was a decrease in n-3 FA in CSF in relation to healthy people. The strongest decrease was observed in the precursor for n-3 FA: linolenic acid (ALA, C18:3 n- 3) and in eicosapentaenoic acid (EPA, C20:5 n-3). The deficit of n-3 FA in cerebrospinal fluid from patiens with neuroinfections when compared to the control was seen also after cluster analysis. ALA is a precursor for n-3 series, including EPA or DHA, they show strong anti-inflammatory effects (Gdula-Argasińska et al. 2016; Wysoczański et al. 2016). Inflammation is an important element of the body’s fight with infection, however, the body also has effective mechanisms to quench the inflammatory reaction. The lack of such a mechanism and an excessive pro-inflammatory reaction would pose a significant threat to the homeostasis of the body. The process of resolution of inflammation itself does not have an immunosuppressive character, as several pro-resolving mediators increase survival from different infections. n-3 FA are one of the elements of such extinguishing, hence the observation in our study. A reflection of the changes in the n-6 and n-3 FA is more than three-fold reduction of n-3/n-6 ratio, this effect was observed for both types of neuroinfections studied.

The changed FA profile in CSF appears to be caused by the synthesis of lipid mediators, including eicosanoids, generated during inflammation by microglia and astrocyte cells. These changes may also be related to the biosynthesis of endocannabinoids, as well as being the result of a diversified expression of genes involved in the synthesis, elongation and desaturation of FA during the inflammatory process (Serhan and Petasis 2011; Gdula-Argasińska et al. 2016). Endocannabinoids are lipid mediators which play an important physiological role via cannabinoid receptor (CB1 and CB2) activation and signalling. Both DHEA and EPEA have anti-inflammatory properties and have been detected in both the brain and retina (Gdula-Argasińska and Bystrowska 2016; Wysoczański et al. 2016; McDougle et al. 2017). In our study we observed the highest content of DHAE, EPEA and NEA in the CSF of patients with VM. In samples from BM patients the ECB level was lower. ECB synthesis during neuroinfection is probably one of the mechanisms by which inflammation is resolved.

The dense clustering of the different cell types during inflammation presents a unique situation for lipid handling. In contrast to the synthesis of protein mediators (cytokines), lipid mediators can be produced along enzyme pathways that involve multiple cells, including microglia, in a process known as trans-cellular biosynthesis (Serhan and Petasis 2011; Norris and Dennis 2014; Johnson 2015). The inflamed tissue becomes a specialized organ for lipid metabolism, producing the types and amounts of lipid mediators needed to promote or resolve inflammation. However, the higher content of long-chain MUFA and PUFA in CSF obtained from patients with BM and VM may be correlated with higher expression of stearoyl-coenzyme A desaturase (SCD1) as well as elongases (Gdula-Argasińska and Bystrowska 2016; Sergeant et al. 2016).

### Changes in fatty acids in CSF as a consequents of BBB permeability

The second mechanism which may explain the results observed in our study is passive FA penetration from the plasma through the damaged BBB as a result of the neuroinfection. Under physiological conditions, the BBB provides effective protection of the nervous system from external factors. The transcellular transport across the BBB involves diffusion and active transport with the use of carrier membrane proteins. The diffusion process transports mainly small-molecule nutrients and fat-soluble substances. Peptides and regulatory proteins as well as protein hormones penetrate the BBB via active transport with protein carriers. The transported substances are not transmitted directly to neurons, but to glial pellets. The astrocyte protrusions cover about 90% of the surface of the capillary walls. These cells constitute the second component, also crucial for the effectiveness of the physical barrier. They are links between the capillary wall and neurons (Ballabh et al. 2004; Nakagawa et al. 2009). The exact characteristics of the BBB are presented in review by Sorge et al. (van Sorge and Doran 2012).

CSF is a secretion of the choroid plexuses located in the wall of the cerebral ventricles and periventricular organs. The transport of substances from the blood to CSF ​​is very selective, because glial cells (tanycetes) adhere to the endothelium and functions to assist in taking certain substances (eg. hormones) from the blood and transferring them to the CSF, as well as in the opposite direction (Lossinsky and Shivers 2004). BBB permeability disorders have been described in various neurological diseases, including inflammatory, infectious, neurodegenerative and neoplastic diseases. A special group of diseases that change the physiological properties of BBB include neuroinfections. Under physiological conditions, there is little infiltration of T-lymphocytes and monocytes through the BBB, the situation changes during infectious processes involving the nervous system, during which an increased transmission of cells of the immune system across the BBB is observed (Weiss et al. 2009). Impaired integrity of the BBB is one of the key factors in BM pathogenesis and is also extremely important for the development of VM. This may be due to the toxic effect of pathogens, their products or through the ability to interact with BBB structures, mainly AJs and TJs. For example, group B *Streptococcus* (GBS) and *Streptococcus pneumoniae* directly affect BBB by producing spore-forming toxins (Nizet et al. 1997; Zysk et al. 2001; Lembo et al. 2010). As GBS produce more toxins, they are more apt to cause BM (Doran et al. 2003). Many viruses damage the cytoskeletal actin during their life cycle (Taylor et al. 2011). In addition, in the course of induced neuroinfections with various etiologies of the immune reaction, cytokines/chemokines molecules are produced that may interfere with the functioning of the BBB, which in turn facilitates the development of neuroinfections and is associated with worsening the prognosis of such patients (van Sorge and Doran 2012). An example is the increase in systemic expression of TNF-α, which translates into an increase in BBB permeability (Sharief et al. 1992). Thus, with the BBB’s excess permeability increasing in the course of neuroinfection, FA could passively pass into CSF.

In our study, there was no significant difference in majority FA between BM and VM groups. But the general trend of changes in both groups with neuroinfection was both the same and with similar intensity. Significant differences were demonstrated for LA, GLA and DHA content.

In conclusion, FA are an important element of the inflammatory reaction in the course of neuroinfection, as demonstrated by the significant increase in n-6 FA and decrease in n-3 FA as well as significant increase of EPEA, DHEA in CSF during neuroinfections of various etiology in relation to healthy people. A limit of our study was that patients were not supplemented with PUFA, therefore it seems necessary to consider this area in the future.

The process of resolution of inflammation, so important to the health of the patient, is still not fully understood. Therefore, the description of fatty acid impaired metabolism in the course of neuroinfection appears to be an important issue. It may allow, inter alia, to develop new pharmacological strategies, therapeutic aims and modern drugs, essential in the fight against neuroinfections. It appears that fatty acids and their metabolites, which are ligands for peroxisome proliferator receptors (PPARs) and nuclear factor kappa B (NF-ĸB) transcription factors (Gdula-Argasińska and Bystrowska 2016; Qasem et al. 2018) may be used as nutraceuticals in the regulation of brain immune response.

## Abbreviations

AA: arachidonic acid
AJs: adherent junctions
BBB: blood–brain barier
BM: bacterial meningitis
CNS: central nervous system
CSF: cerebro-spinal fluid
CRP: C-reactive protein
DHA: docosahexaenoic fatty acid
DHEA: docosahexaenoyl ethanolamide
ECB: endocannabinoids
EPA: eicosapentaenoic fatty acid
EPEA: eicosapentaenoyl ethanolamide
FA: fatty acids
GOS: Glasgow Outcome Scores
GSC: Glasgow Coma Scale
MUFA: monounsaturated fatty acids
NEA: nervonoyl ethanolamide
PUFA: polyunsaturated fatty acids
Q_25_: lower quartile
Q_75_: upper quartile
SFA: saturated fatty acids
TJs: tight junctions
VM: viral meningitis
WBC: white blood cells
